# Surfactant-free synthesis of Cu_2_O hollow spheres and their wavelength-dependent visible photocatalytic activities using LED lamps as cold light sources

**DOI:** 10.1186/1556-276X-9-624

**Published:** 2014-11-22

**Authors:** Yuxi Wang, Da Huang, Xingzhong Zhu, Yujie Ma, Huijuan Geng, Ying Wang, Guilin Yin, Dannong He, Zhi Yang, Nantao Hu

**Affiliations:** 1 Key Laboratory for Thin Film and Microfabrication of Ministry of Education, Department of Micro/Nano Electronics, School of Electronic Information and Electrical Engineering, Shanghai Jiao Tong University, Shanghai 200240, People's Republic of China; 2 National Engineering Research Center for Nanotechnology, Shanghai 200241, People's Republic of China

**Keywords:** Cuprous oxide, Hollow spheres, Surfactant-free, Photocatalysis, LED cold light sources

## Abstract

A facile synthesis route of cuprous oxide (Cu_2_O) hollow spheres under different temperatures without the aid of a surfactant was introduced. Morphology and structure varied as functions of reaction temperature and duration. A bubble template-mediated formation mechanism was proposed, which explained the reason of morphology changing with reaction temperature. The obtained Cu_2_O hollow spheres were active photocatalyst for the degradation of methyl orange under visible light. A self-designed equipment of light emitting diode (LED) cold light sources with the wavelength of 450, 550, and 700 nm, respectively, was used for the first time in the photocatalysis experiment with no extra heat introduced. The most suitable wavelength for Cu_2_O to photocatalytic degradation is 550 nm, because the light energy (2.25 eV) is closest to the band gap of Cu_2_O (2.17 eV). These surfactant-free synthesized Cu_2_O hollow spheres would be highly attractive for practical applications in water pollutant removal and environmental remediation.

## Background

Recently, semiconductor nanomaterials with different morphologies have attracted lots of interests because structure significantly influences their physical and chemical properties. Various morphologies, such as nanowires [1], nanocubes [2], nanocages [3], and octahedrons [4], have been synthesized for their interesting properties and applications. Among these nanostructures, hollow nanostructures are of particular interest because of their unique electrical, magnetic, thermal, and optical properties [5-13]. Hollow nanomaterials are widely used as nanoscale chemical reactors [14], high-performance catalysts [14-16], drug-delivery carriers [17,18], lithium-ion battery materials [19], and wavelength optical components for biomedical applications [20]. According to the reports related to the preparation of hollow materials, various methods have been developed which can be categorized into the following classes: template-mediated approaches [21], chemical etching [22], galvanic replacement [23], and the Kirkendall voiding [6]. Among the above methods mentioned, template-mediated approaches are the most usual and popular ones, which are based on selectively removing the cores in spherical core-shell particles by a solvent or calcination method.

Cuprous oxide (Cu_2_O), a typical p-type semiconductor with a direct band gap of 2.17 eV, has been broadly applied in photocatalysis [24], gas sensors [8,25], solar cells [26,27], photoelectrochemical cells [28,29], and lithium-ion batteries [19]. It is noticed that Cu_2_O with different shapes have attracted much attention. Many efforts have been made to obtain Cu_2_O nanomaterials [30]. Wet chemical reduction [31-35], electrodeposition [11,12,36-38], solvothermal synthesis [39-41], and irradiation [42,43] methods have been applied to prepare Cu_2_O nanocrystals. However, the reported synthetic routes are relatively complex and time consuming, typically involving expensive toxic solvents and surfactants, which make it difficult to purify as-produced Cu_2_O hollow nanostructure as well as produce it in large scale [25,44-46]. Therefore, it is highly rewarding to facile synthesize functional Cu_2_O nanomaterials in a solution without a surfactant.

Meanwhile, Cu_2_O photocatalyst can convert solar into chemical energy to degrade pollutants and can be used as a promising catalyst for environmental wastewater treatment in practical application [24]. Xe lamps and high-pressure mercury lamps with the power of 150 and 400 W, respectively, are usually used as light sources in photocatalytic experiment. They will introduce large amount of heat into the catalytic system, which makes it difficult to control the reaction temperature.

Herein, we investigate Cu_2_O hollow spheres via a facile aqueous solution method under different temperatures without the addition of a surfactant. In our research, hollow spheres with uniform diameter can be obtained through this surfactant-free method. Morphologies of Cu_2_O hollow spheres prepared under different temperatures are displayed and so does the supposed formation mechanism. In addition, photocatalytic activities of Cu_2_O hollow spheres are measured for the first time with a self-designed equipment using light emitting diode (LED) cold lamps with different characteristic wavelengths as photocatalysis light source. LED lamps with the power of 8 W, as typical cold light sources, are different from the high-power Xe lamps and mercury vapor lamps. There is no extra heat introduced into the catalytic system using LED cold light and the wavelength can be easily controlled. This one-pot method proceeds in aqueous medium with low temperatures and high reaction rates, which makes the as-produced Cu_2_O hollow spheres highly attractive for practical applications in water pollutant removal and environmental remediation.

## Methods

### Materials

Copper sulfate pentahydrate (CuSO_4_ · 5H_2_O) and hydrazine hydrate (N_2_H_4_ · H_2_O) are purchased from Sinopharm Chemical Reagent Co., Ltd. (Shanghai, China), of analytical grade, and used without further purification.

### Preparation of Cu_2_O hollow spheres

In a typical synthesis, 0.25 g of CuSO_4_ · 5H_2_O were dissolved in 50 mL of deionized water with continuous stirring. Then, the transparent solution was kept in a 100-mL flask under different temperatures. We used N_2_H_4_ · H_2_O (20%) to reduce Cu^2+^ by fast injection of 1 mL N_2_H_4_ · H_2_O into the solution and stirring at 750 rpm for 1 h. The color of the solution turned from dark blue to brick red with no extra alkali added. After that, the product was centrifuged at 3,250 × *g* for 10 min, washed with deionized water for several times, and finally dried in a vacuum at 60°C for 8 h.

### Photocatalytic activities

Photocatalytic degradation of methyl orange (MO) was carried out in a self-designed equipment. Twenty milligrams of as-prepared Cu_2_O hollow spheres and 50 mL MO solution (10 mg/L) were kept in a 100-mL round-bottom flask with continuous stirring. Four 8-W LED lamps with the same characteristic wavelengths (450, 550, or 700 nm) were used for the first time as cold light sources which were mounted at 10 cm around the solution. Vigorous stirring was employed to ensure the adsorption equilibrium and eliminate any diffusion effect. The MO solution was kept in darkness for 15 min to get adsorption equilibrium and then under visible light.

### Characterizations

The sample sizes and morphologies were investigated using scanning electron microscope (SEM) and transmission electron microscope (TEM). SEM images were performed with a Carl Zeiss Ultra 55 from Carl Zeiss AG, Oberkochen, Germany. TEM images were obtained with a JEOL JEM-2100 TEM operating at 200 kV from JEOL Ltd., Akishima, Tokyo, Japan. The crystal structures were examined by X-ray diffractometer (XRD; D8 Advance, Bruker, Ettlingen, Germany) with Cu Kα (*λ* =1.5418 Å) and 2*θ* from 20° to 80°. Ultraviolet–visible spectra (UV–vis, Lambda 500, PerkinElmer, Waltham, MA, USA) characterizations were carried out at the region from 350 to 600 nm. Nitrogen adsorption-desorption isotherms were collected on an autosorb-iQA3200-4 sorption analyzer (Quantatech Co., New York, NY, USA). The pore size distribution plots were obtained using the Barret-Joyner-Halenda (BJH) model.

## Results and discussion

### Morphology and structure

**Figure 1:**
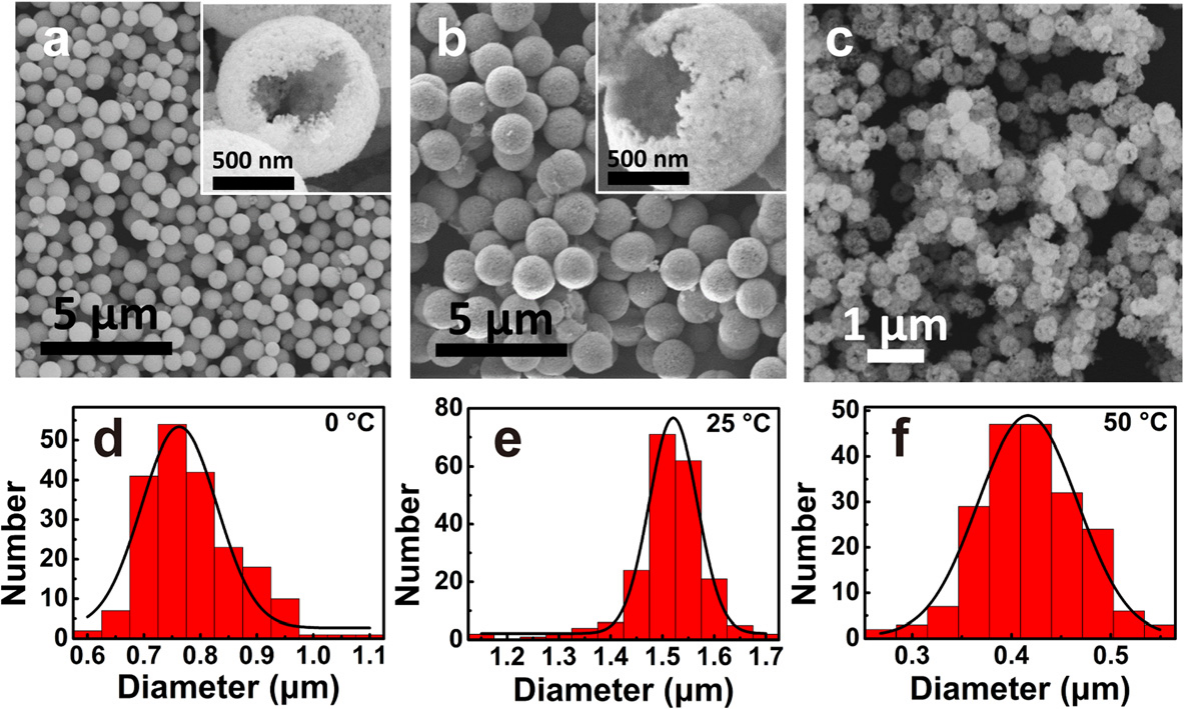
**SEM images and particle size distribution.** SEM images of Cu_2_O hollow spheres obtained at 0°C **(a)**, 25°C **(b)**, and 50°C **(c)**. The insets show the corresponding hollow structures. Diameter distributions of Cu_2_O hollow spheres prepared at 0°C **(d)**, 25°C **(e)**, and 50°C **(f)**.

Uniform Cu_2_O hollow spheres with rough surface were obtained by the simple one-step wet synthesis method. Figure 1 shows SEM images and diameter distributions of Cu_2_O spheres prepared under different temperatures. It can be clearly observed that the sizes and structures of Cu_2_O spheres changed under different conditions. When the reaction takes place in ice water bath keeping at 0°C, the obtained Cu_2_O spheres are well distributed in size with a diameter of 763 ± 83 nm (Figure 1a,d). Few spherical particles are broken into pieces (inset of Figure 1a), so we can clearly find the hollow structure of the big sphere. The big sphere is made up of small particles, leaving nanoscale holes on the surface. At 25°C, the spheres are bigger in size with a diameter of 1,521 ± 73 nm (Figure 1b,e). In addition, the hollow structure can be easily observed from the broken sphere (inset of Figure 1b). However, when the reaction temperature increase to 50°C, the sphere morphology change a lot, which could hardly be called hollow sphere. Cu_2_O spheres prepared at 50°C have rough surfaces with a diameter of 417 ± 51 nm (Figure 1c,f). The reaction time should be strictly controlled within 1 h to prepare Cu_2_O spheres.

**Figure 2:**
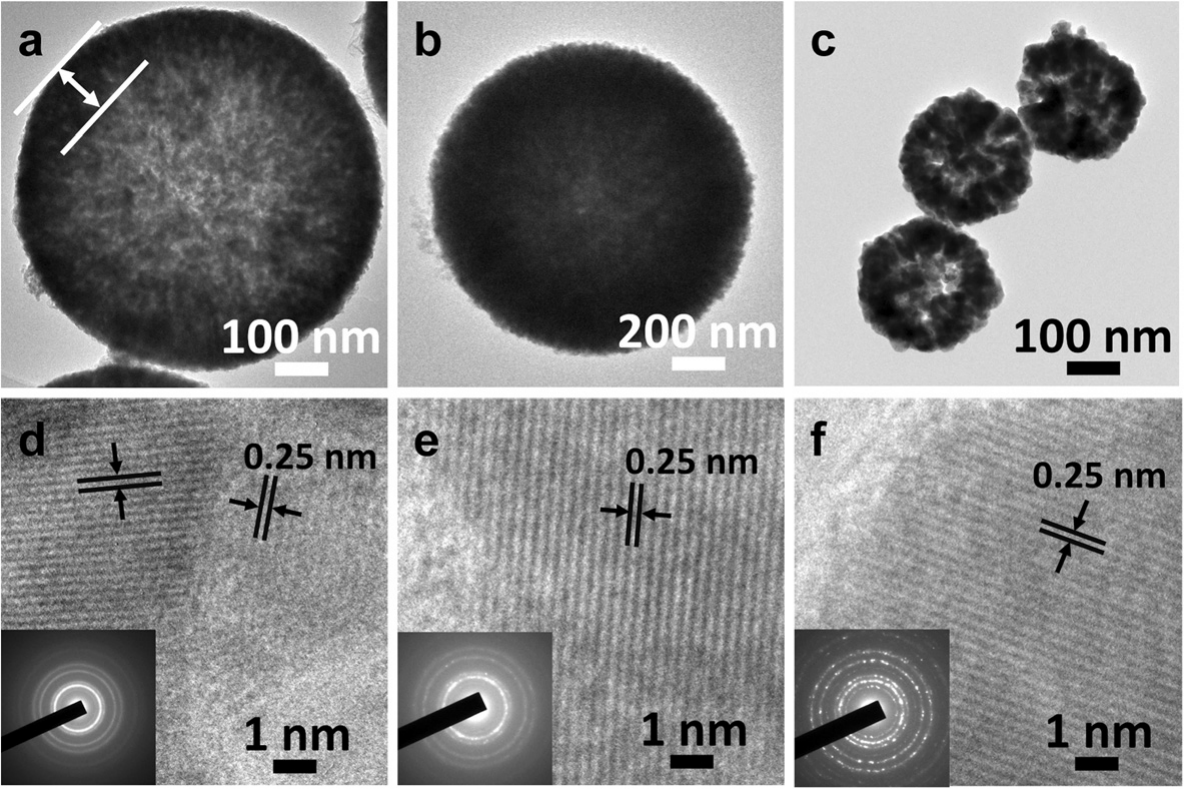
**TEM and HRTEM images.** TEM and HRTEM images of Cu_2_O hollow spheres prepared at 0°C **(a, d)**, 25°C **(b, e)**, and 50°C **(c, f)**. The insets show the corresponding SAED patterns.

Figure 2 shows the TEM, high-resolution TEM (HRTEM), and selected area electron diffraction (SAED) images of Cu_2_O spheres prepared under different temperatures. Figure 2a shows the morphology of Cu_2_O spheres prepared at 0°C in which a hollow structure can be distinctly observed. The obtained Cu_2_O spheres are uniform in size with a wall thickness of 130 nm. The hollow structure shown in Figure 2b can also be observed when the reaction temperature increases to 25°C. Figure 2c shows the morphology of Cu_2_O spheres prepared at 50°C, in which nanoparticles aggregate together to form small sphere-like shape. A fringe spacing of 0.25 nm shown in the HRTEM images (Figure 2d,e,f) corresponds well to that of the lattice space of {111} of Cu_2_O crystals.

**Figure 3:**
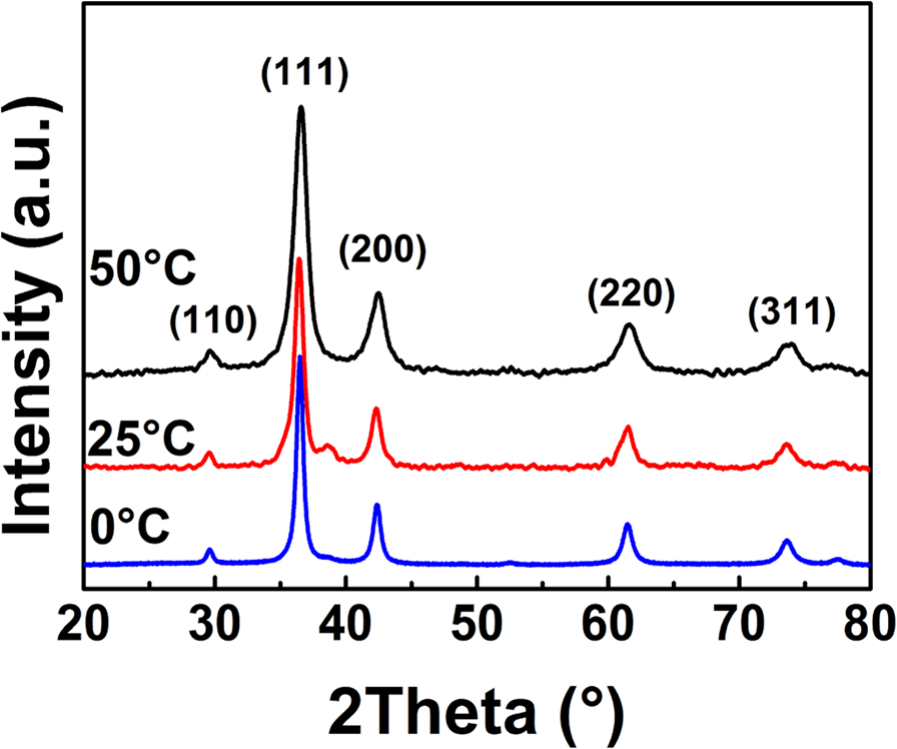
**XRD patterns.** XRD patterns of Cu_2_O hollow spheres prepared at 0°C, 25°C, and 50°C.

The composition and phase purity of the products prepared at different reaction temperatures were characterized by XRD as shown in Figure 3. XRD patterns are confirmed with the SAED results, which show the expected (110), (111), (200), (220), (311), and (222) diffraction peaks corresponding to crystal planes of the Cu_2_O crystals. The insets of Figure 2d,e,f show the SAED patterns of the obtained Cu_2_O spheres, in which the diffraction rings fit well with crystal planes of Cu_2_O. No other peak is observed in the XRD patterns, indicating that the products are phase-pure Cu_2_O crystals. There is no impurity such as cupric oxide or copper.

### Formation mechanism

Hydrazine hydrate (N_2_H_4_ · H_2_O) is used as the reductant to prepare Cu_2_O hollow spheres. As N_2_H_4_ · H_2_O is an alkali reductant with strong reducing ability, after injecting N_2_H_4_ · H_2_O, the solution turns into dark blue within several seconds and then changes to brick red gradually, which means that Cu(OH)_2_ is generated and finally reduced to Cu_2_O. The formation of Cu_2_O hollow spheres in the reaction system can be represented by the following chemical reactions:

$${Cu}^{2+}\;+\;2{OH}^{‒}\;=\;Cu{\left(OH\right)}_{2}$$

$$4Cu{\left(OH\right)}_{2}\;+\;N_{2}H_{4}\;=\;2Cu_{2}O\;+\;6H_{2}O\;+\;N_{2}$$

**Figure 4:**
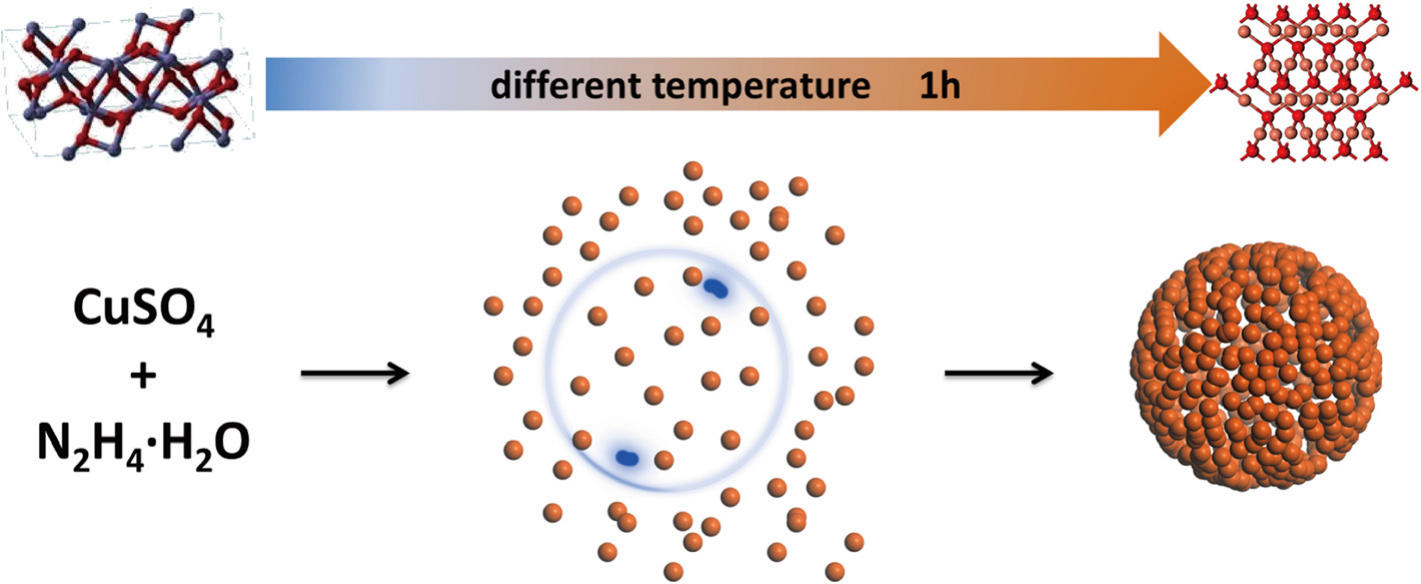
Schematic formation mechanism for bubble template process.

The process of morphology changing under different temperatures can be explained as the following steps in Figure 4.

At a certain temperature, for example, at 25°C, after the addition of N_2_H_4_ · H_2_O into CuSO_4_ solution, Cu^2+^ is reduced to Cu_2_O nanoparticles, and N_2_ nanobubbles are generated at the same time. As there is no surfactant in the reaction system, Cu_2_O nanoparticles will tend to absorb on the surface of N_2_ bubbles, so that Cu_2_O nanoparticles assemble into hollow spheres (Figure 1a), which can be referred to the Ostwald ripening process [47]. When the reaction takes place at 0°C, the reaction rate will slow down, resulting in smaller N_2_ bubbles and spheres with smoother surface and tighter structure, which also agrees with the SEM results and diameter distribution (Figure 1). The reaction rate increases along with the temperature rises. At 50°C, the reaction speed is too high for the nanoparticles to form uniform spheres. In Figure 1c, the hollow sphere structure could hardly be observed. N_2_ nanobubbles escape faster so that the obtained Cu_2_O spheres are smaller.

On the other hand, Cu_2_O spheres are made up of nanoparticles. Crystallization rate increases with the rise of temperature to form bigger nanoparticles, so that the obtained Cu_2_O spheres would have rougher surface, which is also in agreement with the SEM results (Figure 1).

**Figure 5:**
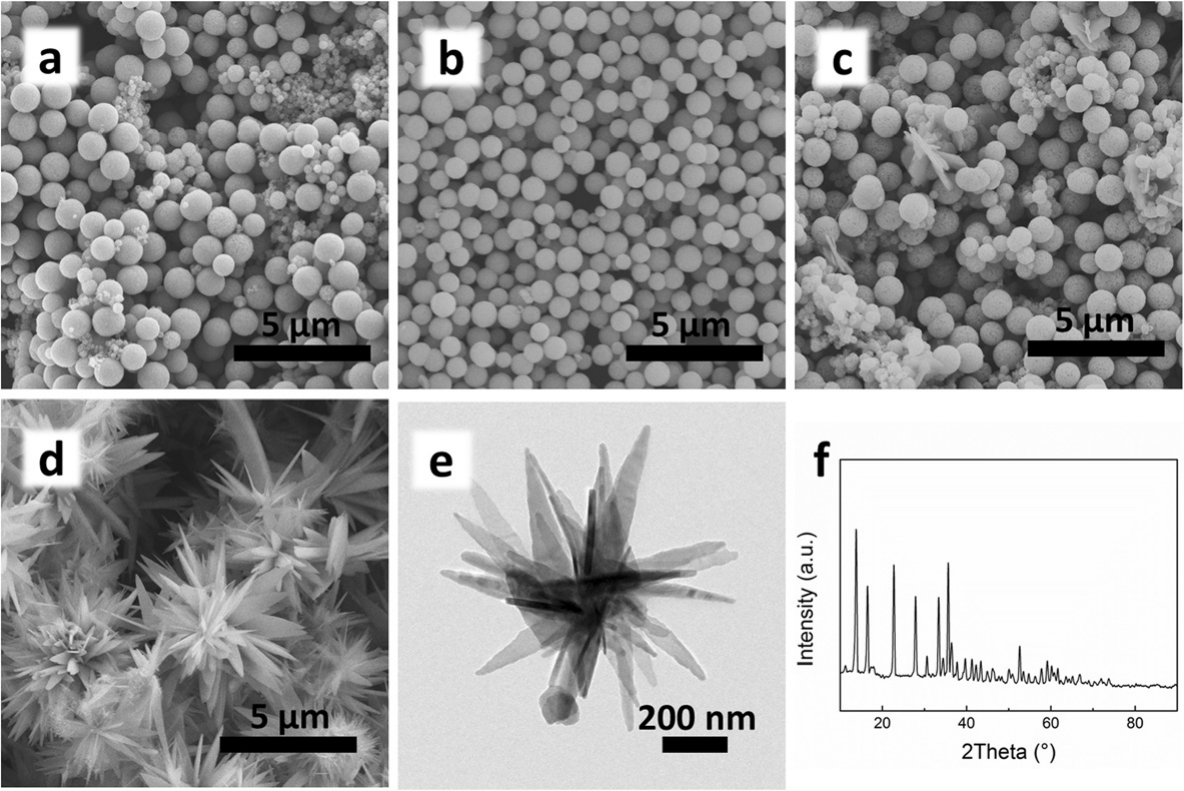
**SEM images of formation process.** SEM images of Cu_2_O hollow spheres obtained at 0°C during different formation times (**(a)** 0.5 h, **(b)** 1 h, **(c)** 2 h, **(d)** 4 h). TEM image **(e)** and XRD pattern **(f)** of Cu_4_(OH)_6_SO_4_.

Meanwhile, the morphology of Cu_2_O hollow spheres changes during the reaction time. The SEM images obtained at 0°C during different formation times are displayed in Figure 5a,b,c,d. Hollow structures have been formed at 30 min but with inconsistent diameters. With the reaction time increase to 6 h, Cu_2_O is oxidized into Cu_4_(OH)_6_SO_4_ by oxygen dissolved in the solution, which can be confirmed by TEM and XRD results in Figure 5e,f.

### Photocatalytic activities

**Figure 6:**
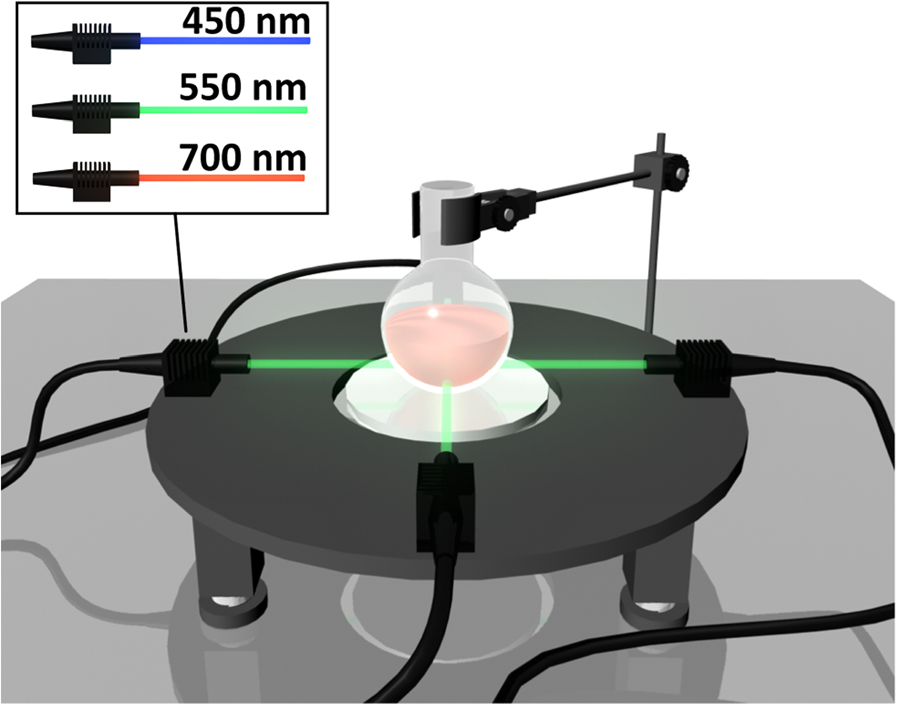
**Schematic diagram of the self-designed photocatalytic equipment setup.** The inset shows the figure of different LED lamps as cold light sources with a wavelength of 450, 550, and 700 nm, respectively.

A self-designed equipment was applied to carry out the photocatalytic degradation experiment as shown in Figure 6. Four 8-W LED lamps were used as cold light sources which were mounted at 10 cm around the center of the reaction round-bottom flask. This equipment can ensure specific wavelength of the light with no heat introduced as LED lamps are cold light sources. We chose LED lamps with different characteristic wavelengths to serve as the light source of photocatalysis for the first time. LED lamps, as typical cold light sources, are different from Xe lamps with the power of 150 W and often used in the photocatalytic experiment. There is no extra heat introduced into the reaction system using LED cold light and the wavelength can be easily controlled. The LED lamps of wavelength 450, 550, and 700 nm were chosen as the visible light sources, as the limited experiment resources we have.

To test the photocatalytic activities of obtained Cu_2_O hollow spheres, MO, a negatively charged molecule, was used in the photodegradation experiments.

**Figure 7:**
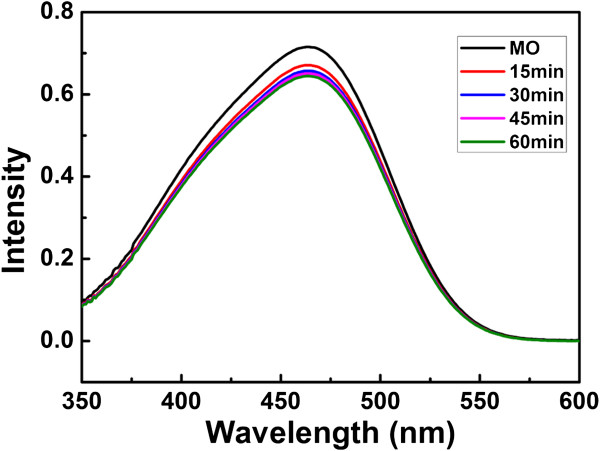
**Adsorption effect of Cu**_**2**_**O hollow spheres prepared at 0°C.** The MO solution was kept in dark environment.

**Figure 8:**
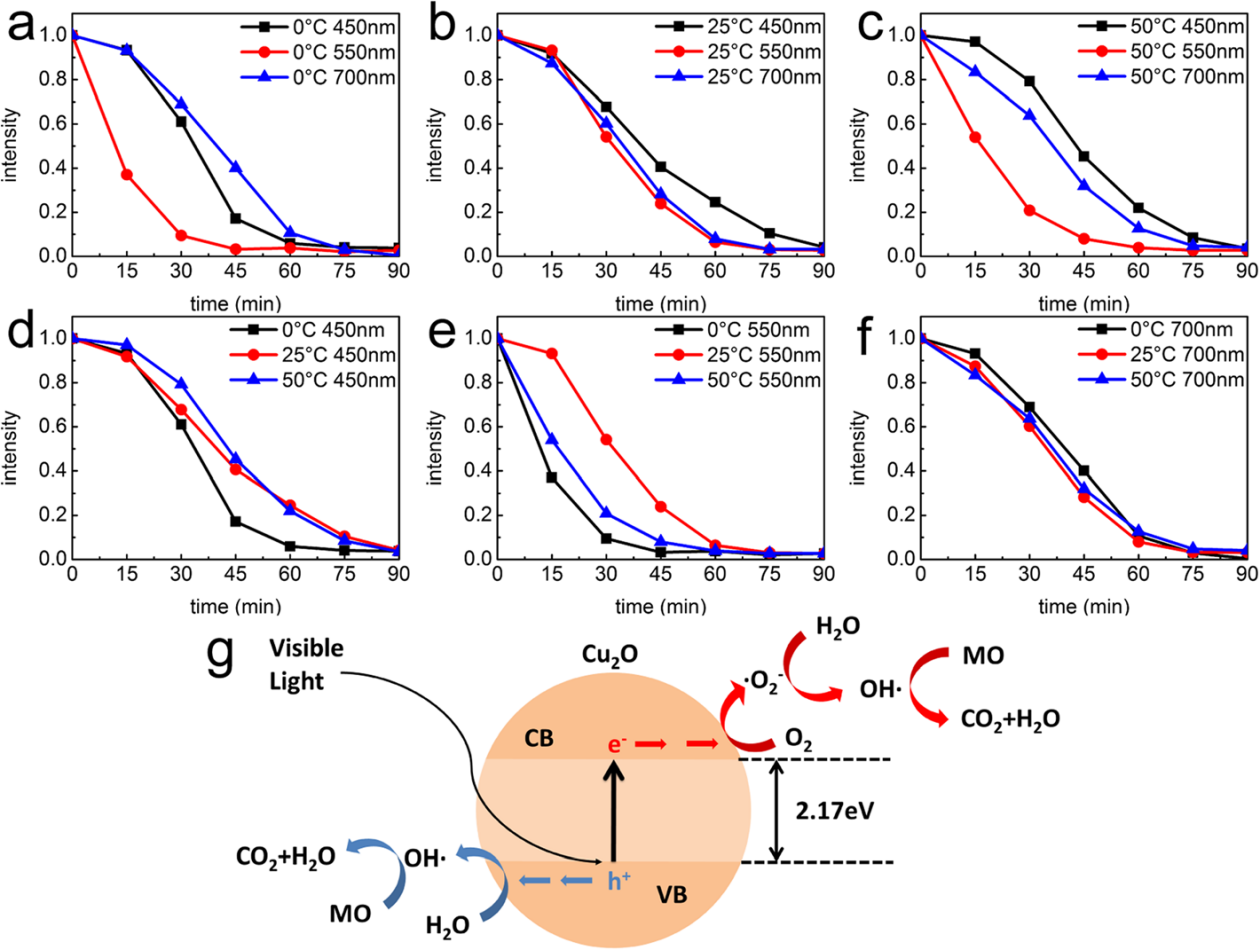
**Degradation of MO in the presence of Cu**_**2**_**O hollow spheres.** Degradation of MO in the presence of Cu_2_O hollow spheres prepared at different temperatures irradiated by different wavelengths of visible lights **(a-f)** and photocatalytic mechanism **(g)**.

MO solution was kept in darkness for 15 min to get adsorption equilibrium. The adsorption curve in darkness is shown in Figure 7. It is found that adsorption have reached equilibrium after 15 min. Figure 8a,b,c,d,e,f shows the photocatalytic performance of Cu_2_O as photocatalysts for the degradation of MO. The experimental results disclose that Cu_2_O hollow spheres allow superior photocatalytic activity. Meanwhile, the time for concentration of MO solution to reach 1/*e* is summarized in Table 1, so we can have a clear look at the degradation of MO.

**Table 1:** **Time for the intensity of MO to achieve 1/*e***

**Wavelength (nm)**	**Energy (eV)**	**Time to achieve 1/*e *** **(min)**		
		**0**°**C**	**25**°**C**	**50**°**C**
450	2.75	28.27	48.67	50.49
550	2.25	15.03	33.64	22.86
700	1.77	46.67	40.09	42.67

The results can be explained in two aspects, different wavelengths of visible lights and photocatalysts prepared at various temperatures. As shown in Figure 8a,b,c, the photocatalytic results indicate that the obtained Cu_2_O spheres can photocatalyze MO degradation under visible light and the 550-nm wavelength light exhibits the most effective photocatalytic effect among all three spheres.

The light energy can be calculated using Einstein's photoelectric effect equation as follows:

$$E=h\frac{c}{\lambda }$$

**Figure 9:**
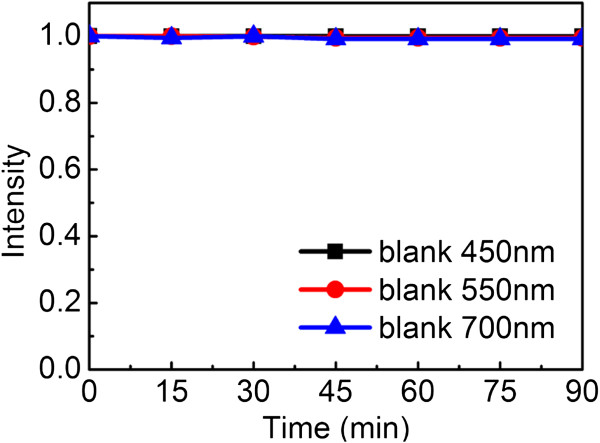
**Contrast results without Cu**_**2**_**O hollow spheres.** The MO solution was kept in the same photocatalytic conditions without Cu_2_O hollow spheres.

In the equation, *E* represents the energy of light, *h* is the Planck's constant which equals 6.626 × 10^-34^, *c* is the velocity of light, and *λ* refers to the experimental wavelength. When the photocatalytic reaction takes place under light of 550-nm wavelength, the energy is about 2.25 eV, which is very close to the band gap of Cu_2_O (2.17 eV). Therefore, 550 nm is the most suitable wavelength for Cu_2_O to photocatalytically degrade MO. At the same time, a contrast experimental result indicates that the MO solution will not be degraded without Cu_2_O hollow spheres under different wavelengths (Figure 9).

**Figure 10:**
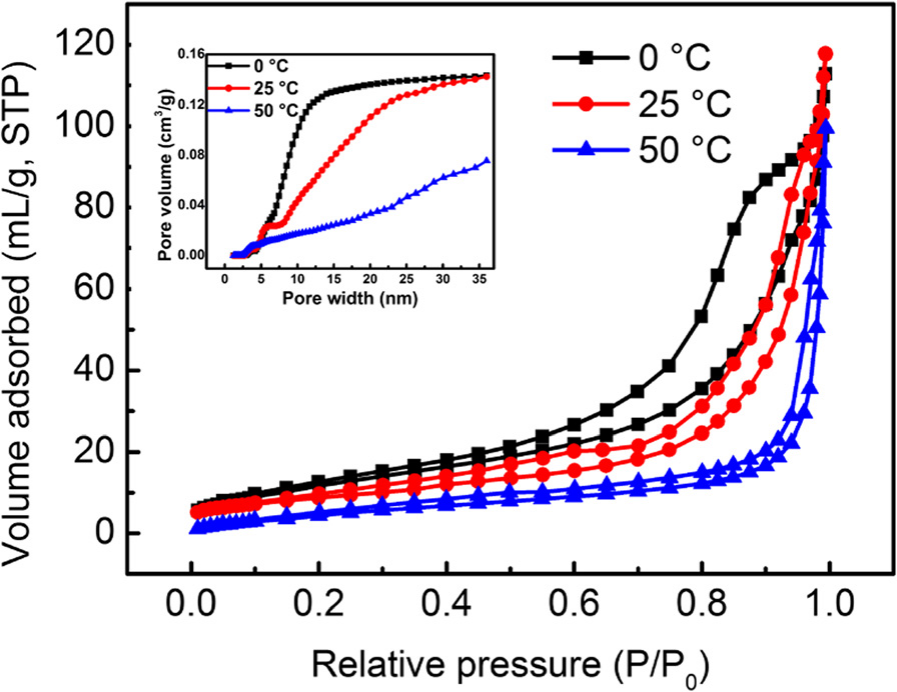
**N**_**2 **_**adsorption-desorption isotherm curves.** N_2_ adsorption-desorption isotherm curves of the different samples prepared at 0°C, 25°C, and 50°C. The inset shows the pore size distributions of the different samples.

As for different wavelengths of visible lights (450 and 550 nm), Cu_2_O hollow spheres prepared at 0°C exhibit the highest degradation effect (Figure 8d,e), because Cu_2_O spheres prepared at different temperatures possess different Brunauer-Emmett-Teller (BET) surface areas. N_2_ adsorption-desorption isotherms (Figure 10) and the corresponding BJH pore size distribution plots (inset of Figure 10) of the obtained Cu_2_O spheres are performed. It can be seen that the Cu_2_O spheres prepared at 0°C have a relatively narrow pore size compared to the other two samples. Moreover, the BET surface area of the prepared Cu_2_O spheres under 0°C (45.985 m^2^/g) is larger than the surface areas of Cu_2_O spheres under 25°C (31.961 m^2^/g) and under 50°C (20.944 m^2^/g). The larger BET surface area of the Cu_2_O crystals can be attributed to the hollow structure and interconnected pores in the crystals.

The structure with larger BET surface area could facilitate effective contacts between Cu_2_O spheres and organic contaminants, enhancing light harvesting and ultimately improving the photocatalytic activities. However, it shows almost the same effect under 700-nm wavelength among the three kinds of Cu_2_O spheres (Figure 8f). Maybe under 700-nm wavelength LED lamps, the structure of spheres is not the dominant factor of the photocatalytic activities.

An illustration of inter-particle electron transfer behavior is proposed as shown in Figure 8g. The uniform distributions of Cu_2_O hollow spheres have large active surface area, which enhances the effective adsorption of photons and provides a continuous pathway for the transportation of photoinduced electrons. The electrons in the valence band of Cu_2_O are excited to its conducting band, giving rise to the formation of electron and hole pairs. The obtained electrons and holes with high energy can combine with H_2_O and reduce MO into CO_2_ and H_2_O.

## Conclusions

We demonstrate a facile method to prepare Cu_2_O hollow spheres. Under the preparation at 0°C, 25°C, and 50°C, the obtained Cu_2_O hollow spheres have diameters of 763 ± 83, 1,521 ± 73, and 417 ± 51 nm, respectively. The corresponding surface area is 45.985, 31.961, and 20.944 m^2^/g, respectively. Cu_2_O hollow spheres are obtained by nanoparticles absorbing on the surface of N_2_ bubbles and assemble together. A bubble template process is introduced to explain the formation mechanism. Importantly, Cu_2_O hollow spheres exhibit better photocatalytic activities for MO degradation under visible light. This is because the developed BET surface areas lead to more contact points, thus forming much more active sites between MO and the catalyst. So, Cu_2_O hollow spheres prepared at 0°C are the most effective for the degradation of MO. At the same time, 550 nm is the most suitable wavelength for Cu_2_O to photocatalytically degrade MO, because the light energy (2.25 eV) is closest to the band gap of Cu_2_O (2.17 eV).

The work not only provides insights into the Cu_2_O catalysis but is also useful for better catalyst design and water treatment industry. The LED lamps as cold light sources with no extra heat introduced into the reaction system are promoted in this work. In summary, we provide an efficient synthetic strategy for the fabrication of effective Cu_2_O visible photocatalyst in environmental treatment, and the self-designed catalytic equipment with single-wavelength LED cold light sources exhibits a novel model for the catalytic design.

## Competing interests

The authors declare that they have no competing interests.

## Authors’ contributions

YW performed most of the experiment and wrote the manuscript. DH and XZ helped analyze the characterization results. YM, HG, and YW maintained the self-designed photocatalytic equipment. GY and DH characterized the TEM and BET. ZY and NH supervised all of the study and provided financial support. All authors read and approved the final manuscript.
